# RF‐EMF Risk Perception and Trust in Radiation Protection Authorities: A Comparative Study on Precautionary Information in Germany and Greece

**DOI:** 10.1002/bem.70042

**Published:** 2026-01-07

**Authors:** Marie Eggeling‐Böcker, Efthymios Karabetsos, Maria Christopoulou, Sarah C. Link, Ferdinand Abacioglu, Christoph Boehmert

**Affiliations:** ^1^ Department for Social Sciences IU International University of Applied Sciences Erfurt Germany; ^2^ Greek Atomic Energy Commission (EEAE) Athens Greece; ^3^ Aalen University Aalen Germany

**Keywords:** mobile communications, precautionary information, radio‐frequency electromagnetic fields (RF‐EMF), risk perception, trust

## Abstract

This study investigates how different types of precautionary information affect risk perception and trust in national radiation protection authorities regarding radio‐frequency electromagnetic fields (RF‐EMF) from mobile communications, with a specific focus on 5G networks. A total of 2169 participants (1040 in Germany, 1129 in Greece) were randomly assigned to one of three conditions: (1) basic information, (2) simple precautionary information regarding possibilities to reduce personal RF‐EMF exposure while using a mobile phone, and (3) conceptual precautionary information, including an explanation distinguishing “precaution” from “prevention” (1 × 3 factorial design). Contrary to the expectation that simple precautionary messages lead to higher risk perception and lower trust compared to basic messages, this was only the case for general conditional risk perception assuming that no precautions are taken, but not for affective risk perception, trust, or general conditional risk perception assuming that precautions are taken. Notably, providing a more elaborate explanation of the precaution/prevention distinction did not decrease risk perception or increase trust compared to giving simple precautionary information only, and even increased risk perception compared to basic information. This suggests limited benefit in emphasizing this conceptual nuance of precaution. Considering other variables, precautionary information increased feelings of self‐efficacy and perception of message consistency. The findings reveal significant country differences: Greek participants reported higher perceived risks and lower trust than German participants. Gender differences also emerged, with women expressing higher risk perception and less trust than men. In contrast to the previous literature, the results suggest that precautionary information concerning personal mobile phone use can be communicated without leading to higher public concern about RF‐EMF exposure from mobile communications. However, we found some evidence that adding conceptual explanations to precautionary information leads to higher risk perception. The results also show that considering sociocultural and individual differences in risk communication can be relevant. Possible explanations for the findings and implications for risk communicators are discussed.

## Summary

- The study investigated risk perception and trust in authorities regarding RF‐EMF from mobile communication, particularly 5G NR networks.
- Precautionary information did mostly not lead to higher risk perception or lower trust, but increased feeling of self‐efficacy. Unexpectedly, adding conceptual information intended to explain the precautionary approach actually increased risk perception.
- Greek (compared to German) and female (compared to male) participants reported higher RF‐EMF risk perception and lower trust in authorities.

## 1 Introduction

Radio‐frequency electromagnetic fields (RF‐EMF) from mobile communications, including the 5G New Radio (NR) networks, have triggered public debates and precautionary policy responses in various countries, even though there are no established health risks (Rowley and Mazar 2021; Stam 2017; World Health Organization WHO 2017). Germany and Greece, for instance, have both seen public concern about exposure to RF‐EMF, though the level and nature of these concerns differed substantially between the two countries (TNS Opinion & Social 2010). For professionals working in radiation protection and risk communication, understanding public perceptions of potential RF‐EMF risks is essential for contextualizing societal responses and developing effective, evidence‐based communication strategies (Lundgren and McMakin 2018). Effective risk communication requires not only scientific and technological expertise, but also knowledge of how the public perceives and emotionally responds to emerging technologies. Risk perception is a key determinant of public acceptance of new technologies (Brauner et al. 2024; Gupta et al. 2012) and is closely related to trust in regulatory institutions (Siegrist et al. 2010). This study examined different communication strategies regarding RF‐EMF from mobile communications, focusing on the provision of precautionary information.

### 1.1 Risk Perception and Trust

Risk perception generally refers to the subjective evaluation of a (potential) hazard and can vary between individuals. It is shaped by cognitive estimations, such as the perceived probability of a hazard occurring and its potential severity (Sjöberg et al. 2004; Wilson et al. 2019) as well as by affective responses (Loewenstein et al. 2001; Wilson et al. 2019). Some theoretical models conceptualize risk perception as multidimensional, highlighting for example dimensions such as affective reactions (e.g., fear or worry), perceived likelihood of exposure, the expected consequences of exposure, and the severity of those consequences (Walpole and Wilson 2021; Wilson et al. 2019).

Studies on risk perception of RF‐EMF used by older generations of mobile communications technology typically report mean values of around the scale mid‐point in the general population (e.g., Siegrist et al. 2005 for Switzerland; White et al. 2007 for the United Kingdom). The percentage of survey respondents that perceives a risk has typically been reported to be between 13% (Kowall et al. 2012) for mobile phones in a German sample and 48% (TNS Opinion & Social 2010) for EMF in general, not specifically mobile phones, in a European Union sample. Surveys on risk perception of RF‐EMF associated with 5G specifically reported comparably higher mean values of about 7 on a scale from 1 to 10 (Koh et al. 2020) and about 60 on a scale from 0 to 100 (Frey 2021). However, all figures above need to be interpreted with caution, considering the method used, that is survey questions. First, comparative risk perception surveys show that figures for other risks such as air pollution are typically higher (Frey 2021; Infas Institut 2005, 2006, 2010; Koh et al. 2020; LINK Institut 2013). Second, it has been shown that among those who perceive RF‐EMF as a risk according to their answers in a survey, RF‐EMF is only a matter of everyday relevance for one in three people (Wiedemann et al. 2017). Consequently, risk perception figures from surveys should not be overinterpreted as a sign of massive concern among the surveyed populations.

Risk judgments (as the perception of scientific information in general) may also be related to trust in sources communicating information about a topic or in a system (e.g., a specific institution, public health protection or science in general). Earle (2010) defines trust as “the willingness, in the expectation of beneficial outcomes, to make oneself vulnerable to another based on a judgment of similarity of intentions or values.” Trust involves risk, is important in situations where there is some kind of (perceived) uncertainty and is often researched alongside risk perception (Siegrist 2021). For citizens thinking about “RF‐EMF/5 G and health,” risk perception and trust may be closely related to each other and have been found to correlate in previous studies (e.g., Boehmert et al. 2017).

### 1.2 Relations to Sociodemographic Factors

Demographic variables such as gender, age, and cultural background can be related to risk perception and trust and are considered in this study. With regard to gender, an extensive body of research suggests that women and men differ in their perception of technological risks, with women reporting higher risk perception than men (Davidson and Freudenburg 1996; Gustafsod 1998). Regarding RF‐EMF from mobile phones and base stations, a correlation was found between gender and a technological risk perception factor identified by Siegrist et al. (2005). This factor included the perception of six technological risks, with mobile phone RF‐EMF risk perception and base station RF‐EMF risk perception showing the highest loadings.

Considering cultural background, this study compares two countries with different public attitudes and regulatory approaches regarding RF‐EMF from mobile communications: Germany and Greece. In Germany, public concern about health effects related to RF‐EMF, including 5G, exists (Dilkova‐Gnoyke et al. 2022), and as in many other countries (World Health Organization WHO 2017), regulatory authorities follow exposure limits recommended by the International Commission on Non‐Ionizing Radiation Protection (ICNIRP 1998, 2020). German authorities have emphasized transparency through public information campaigns (e.g., the German mobile communications research program “Germany talks about 5G” [Bundesregierung Deutschland 2024]). While some activist groups demand stricter limits (e.g., Diagnose Funk 2025), the general governmental approach is to balance technological progress with health protection (Bundesamt für Strahlenschutz 2025). In Greece, public skepticism is more pronounced, for example, in a 2018 nationwide survey by the Greek Atomic Energy Commission (EEAE 2023), widespread concern about radiation‐related issues regarding mobile communications in the Greek population was documented. Governmental precautionary approaches are stricter, which is particularly shown by the implementation of exposure limits that are lower than international recommendations (Stam 2018).

According to the Eurobarometer survey (TNS Opinion & Social 2010), only a minority of German respondents (26%) considered mobile phones to strongly affect health. In Greece, a high proportion of participants perceived health risks from base stations (63%) and mobile phones (53%). Public trust in governmental and EU institutions in Greece is comparatively low, which is also shown in the Eurobarometer survey (TNS Opinion & Social 2010) where 75% of Greek respondents believed they were not adequately protected by public authorities from potential health effects of EMF (EU average: 58%), and 67% expected the EU to provide more information on these risks (EU average: 48%). In Germany, about 48% of the respondents were dissatisfied with available EMF information, a proportion around the EU average and lower than in Greece (54%). Additionally, compared to Greece, fewer German citizens believed that authorities failed to protect the adequately from potential EMF risks, and trust in authorities even showed an improving trend between 2006 and 2010 (−13 percentage points).

The rollout of 5G NR in Germany is not perceived much more critical than previous mobile communications standards by the public (Link et al. 2025). This aligns with Vaupotič's observation that 5G is largely seen as a successor to 3G and 4G rather than as a disruptive new technology (Vaupotič et al. 2025) and with Dilkova‐Gnoyke's (2022) similar conclusion. In Greece, the potential risk for 5G systems was perceived by 35% of the population as high or very high (which is lower than the perceived risk for cell towers cell phone radiation, with 42% and 45%, respectively) (Perko 2025). In the same study, the confidence in authorities for risk management to 5G systems, cell towers and cell phone radiation was perceived as “very much” and “quite a lot” by only almost 20% of the public.

Summarized, these findings suggest that there are large national differences in RF‐EMF and 5G risk perception, which may be shaped by broader cultural differences, trust in institutions or authorities, and historical communication practices. For risk communication strategies to be effective, they must take these sociocultural differences into account. However, one needs to be aware that even though concerns about RF‐EMF from mobile communication technology are present, most people in both Germany and Greece own a mobile phone (Statista 2025a, 2025b).

### 1.3 Precautionary Information

In addition to general information on RF‐EMF, 5G NR, and health, many national authorities, such as the Federal Office for Radiation Protection (BfS) in Germany and the Greek Atomic Energy Commission (EEAE), also provide precautionary information (Bundesamt für Strahlenschutz 2024; Greek Atomic Energy Commission EEAE 2020). The information given can refer to officially implemented safety measures, like precautionary exposure limits (institutional precautionary information), or to voluntary behavioral measures that individuals can adopt to reduce personal exposure, for example, using headsets, keeping distance from the body, or switching off wireless functions when not needed (personal precautionary information).

The communication of precautionary information regarding RF‐EMF used in mobile communications has been the subject of critical debate. Several experimental studies have found that presenting such information can lead to a heightened sense of risk compared to basic information alone (see Boehmert et al. 2020 for a meta‐analysis) and may even reduce trust in public health authorities (Wiedemann et al. 2006; Wiedemann and Schütz 2005). These effects have often been observed in studies that presented brief or institutional precautionary information (Wiedemann et al. 2006; Wiedemann and Schütz 2005). Studies focusing on personal precautionary information have produced more nuanced results. In some cases, no increase in perceived risk was observed (Cousin and Siegrist 2010), or effects were only present in specific subgroups, for example, individuals with low trait anxiety (Boehmert et al. 2017, 2018). Relatively little is known about the psychological process that is involved in this effect. Qualitative evidence suggests that communicating precaution while at the same time stating there is no evidence for any health effects can be perceived as inconsistent or contradictory (Timotijevic and Barnett 2006). Moreover, participants in that study thought it was unlikely that a government (UK government in that case) would take a precautious stance (Timotijevic and Barnett 2006), implying a low trust in this authority.

### 1.4 Precaution and Prevention

When decisions are made, the concept of precaution shows strong similarities to the concept of prevention (Stirling and Tickner 2004). Both are directed towards avoiding potential harm. Unlike prevention, which refers to actions taken when a risk is known and scientifically established, precaution refers to protective measures when there is no proven risk, but scientific uncertainty may still exist (European Environment Agency 2024; Mieg 2022). If applying precaution, the avoidance of potential harm occurs at an even earlier stage than prevention (Weed 2004). The earliest form of prevention, known as primary prevention—and therefore the closest to precaution—addresses the very roots of a risk, for instance discouraging individuals from taking up smoking in order to lower their likelihood of developing lung cancer (Stirling and Tickner 2004). In this example, while there is uncertainty at the level of the individual (whether a particular person will in fact develop lung cancer from smoking is uncertain), there is very little uncertainty at the population level: Smoking is strongly associated with an increased risk of lung cancer. Precaution, by contrast, involves preventive action in situations of uncertainty pertaining not only to the manifestation of a risk at the individual level, but also to the population level, at which there is less evidence for the relation of a specific agent (e.g., EMFs) to adverse outcomes (Weed 2004). Put differently, precaution entails the application of preventive measures in contexts where it remains unresolved whether the agent in question constitutes a hazard at all, but when there are “reasonable grounds for concern” (Commission of the European Communities 2000).

The current study examines whether communicating this conceptual distinction diminishes the effect of precautionary information on risk perception and trust. The underlying idea is that such a clarification could reduce perceptions of inconsistency.

### 1.5 Research Questions and Hypotheses

Given the mixed findings on the effects of precautionary information, the current study examines the psychological effects of different kinds of precautionary communication. This approach aims to identify how different types of precautionary communication affect RF‐EMF risk perception and trust in radiation protection authorities. The hypotheses on the effects of precautionary information address two central research questions:

1. What is the effect of simple precautionary information (compared to basic information only) on risk perception and trust in state authorities of radiation protection?
2. What is the effect of conceptual precautionary explanations (compared to simple precautionary information) on these same outcomes?

The hypotheses were pre‐registered on the Open Science Framework: https://osf.io/b4cms. For the paper, the wording from the pre‐registration was changed in some places, but the contents of the hypotheses stay the same.

(Effects of simple precautionary information vs. basic information):

- **H1a.** Affective risk perception will be significantly higher after reading the simple precautionary text compared to the basic text.
- **H1b.** Conditional risk perception without precautionary behavior (CR1) will be significantly higher in the simple precautionary condition.
- **H1c.** No significant difference is expected in conditional risk perception when precautionary measures are taken (CR2).

Trust in state radiation protection authorities will be significantly lower in the simple precaution condition compared to the basic information condition.

(Effects of conceptual precautionary information vs. simple precautionary information):

- **H3a.** Affective risk perception will be significantly lower after reading the conceptual precautionary information text compared to the simple precautionary text.
- **H3b.** CR1 will be significantly lower in conceptual precautionary information condition.
- **H3c.** No significant difference is expected in CR2.

Trust in state radiation protection authorities will be significantly higher in the conceptual precautionary information condition compared to the simple precautionary information condition.

Furthermore, we compare data from Germany and Greece, demographic variables such as gender and age, as well as personal relevance of the topic “RF‐EMF and health.” By combining communication strategy testing, with cross‐national comparison this study contributes to a better understanding of how RF‐EMF information can be communicated responsibly and effectively in diverse public contexts, focusing on most recent technological developments (5G NR).

## 2 Materials and Methods

### 2.1 Sample

To determine the necessary sample size, a power analysis using the tool G*Power was conducted. The required sample size was calculated for an ANOVA with three groups, a power to be achieved of 0.8, a significance level of 0.05 and the aim to detect small effects (Cohen's *f* = 0.1) was *N* = 969. This was rounded up to *N* = 1000 participants per country and the final sample after exclusions consisted of *N* = 2169 participants, including *n* = 1040 participants from Germany and *n* = 1129 from Greece. The number of participants per country and condition can be seen in Table 1.

**Table 1:** Number of participants per country and condition.

Condition	Germany (*n*, %)	Greece (*n*, %)
Total	1040 (100%)	1129 (100%)
Basic information	349 (33.6%)	362 (32.1%)
Simple precautionary information	356 (34.2%)	371 (32.9%)
Conceptual precautionary information	335 (32.2%)	396 (35.1%)

To obtain a heterogenous and approximately representative sample, interlocking quotas for age and gender as well as a non‐interlocking quota for region were applied. Age was categorized into five groups (18–29, 30–39, 40–49, 50–59, and 60+), and gender was recorded as male or female. Quotas were based on demographic distributions from Eurostat (Eurostat: EU Statistical Agency 2024). During the final phase of data collection, it proved particularly difficult to fulfill quotas for older adults, especially women, in Greece. As a result, quotas for these age groups were adjusted. This slight underrepresentation of older women in the Greek sample should be considered when interpreting results. Recruitment and exclusions are summarized in Table 2.

**Table 2:** Recruitment and exclusions of the participants.

	Germany	Greece
Clicked on the survey link	2404	3644
Did not start participation	49	74
Quota already full	794	1372
Inactivity > 30+ minutes or interrupted response	95	111
Speeding (< 3 min total response time)	106	63
Failed attention checks	313	892
Planned exclusions (sum)	1357	2512
Missing data (soft launch only)	2	2
Gender “diverse” or “other” (excluded)	5	1
Final sample	1040	1129

### 2.2 Survey Preparation and Data Collection

Prior to the start of the study, which was conducted in form of a survey, an ethics application was positively evaluated by the ethics committee of IU International University of Applied Sciences in Germany. The survey was originally developed in German and translated into Greek by a professional translation service. Native Greek‐speaking experts in the field of RF‐EMF reviewed the translation to ensure accuracy and content validity. Pretests were conducted in both languages to evaluate participants' initial reactions, identify unclear phrasing or misunderstandings, and improve usability. A panel provider (Bilendi) was commissioned to recruit the participants; panel members were eligible to participate if they were at least 18 years old and had not participated in prior studies on mobile communications RF‐EMF conducted by the same research team. Participants were contacted via email and received a link to the online survey. They were incentivized through the panel provider's established reward system (e.g., points or monetary compensation). Data collection took place in Germany and Greece in March and April 2024. The survey was designed to take no more than 10 min to complete. Average completion time was *M* = 7.8 min (SD = 4.3) in Germany and *M* = 7.7 min (SD = 4.3) in Greece.

### 2.3 Conditions and Survey Procedure

The study followed a between‐subjects experimental design with one factor: Text type. Participants were randomly assigned to one of three groups:

- Group 1 (Basic information) received a short text explaining what RF‐EMF are, how mobile communications work, and that there is no evidence of adverse health effects below legislated exposure limits, although some scientific uncertainties remain.
- Group 2 (Simple precautionary information) received the same basic text, followed by simple precautionary information on how individuals can reduce personal exposure to RF‐EMF from mobile devices.
- Group 3 (Conceptual precautionary information) received all the information above, plus an explanation of the concept of precaution, including a comparison with prevention.

All texts were based on publicly available material from the German Federal Office for Radiation Protection (BfS) and the Greek Atomic Energy Commission (EEAE) but were shortened and adapted for use in a brief experimental context. To avoid pre‐existing attitudes toward real institutions or authorities from influencing responses, the information in all conditions was presented as coming from a “German/Greek Radiation Protection Agency” instead of providing the actual names of these agencies.

**Figure 1:**
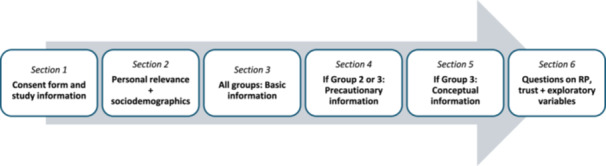
Procedure of the survey.

An overview of the procedure of the survey can be seen in Figure 1, the complete survey can be downloaded from https://osf.io/p9whn/. Participants first read the study information and provided informed consent. Subsequently, they were asked about the personal relevance of the topic “RF‐EMF and health.” Participants were then presented with the information texts, depending on their assigned condition. A 30‐s timer was implemented on each text screen to ensure sufficient reading time before continuation was allowed. After reading the texts, participants answered a set of questions, primarily addressing risk perception and trust in state authorities of radiation protection. Finally, they received a debriefing and were thanked for their participation. All survey questions were mandatory. To ensure data quality, participants encountered two attention checks, if they failed one, they were excluded from the study and data analysis. In the first one, directly after the text, participants were asked: “Which term was abbreviated with ‘EMF’ in the text?.” Only “Electromagnetic Fields” was correct; distractor options were plausible but incorrect. In the second one, within the trust‐related item block, participants saw the instruction: “Please check the box ‘strongly agree.’”

### 2.4 Measures

#### 2.4.1 Risk Perception

Risk perception was captured using self‐report items regarding participants' subjective perceptions. Affective risk perception was measured using three items each for mobile phones and mobile phone base stations, based on Walpole and Wilson (2021). Participants rated how concerned, worried, and afraid they felt about “the EMF emitted by your mobile phone” and “EMF emitted by mobile phone base stations” on a 7‐point Likert scale (1 = not at all, 7 = very much). The items were combined into a single score for analysis.

Risk perception measures are usually unconditional, in the sense that they do not account for the fact that people's risk perception depends on their risk‐related behavior (e.g., Ronis 1992; Van Der Velde et al. 1996). Van Der Velde et al. (1996) suggested measuring risk perception more often conditionally, that is depending on risk‐related behavior. For instance, people's sense of being at risk for sexually transmittable diseases very much depends on whether they use condoms or not. With unconditional risk perception measures, it remains unclear to what extent respondents reflect protective behavior in their ratings. Boehmert et al. (2016) have argued that when investigating the effects of precautionary communication regarding RF‐EMF from mobile communications, it can be particularly important to assess conditional risk perception. Indeed, in that study, individuals rated the risk much higher under the condition that no precautions were taken than under the condition that precautions were taken (Boehmert et al. 2016). Moreover, effects of various versions of precautionary information on risk perception were also more pronounced for conditional risk perception without taking precautions than for an unconditional risk perception measure in that study. In the current study, conditional risk perception was assessed in accordance with Boehmert et al. (2016) under two conditions:

- CR1 (no precautionary measures taken): “How dangerous do you think EMF from mobile phones are if you do not take any precautions?”
- CR2 (precautionary measures taken): “How dangerous do you think EMF from mobile phones are if you take measures to (a) reduce usage duration (e.g., keep calls short), and (b) increase distance (e.g., use a headset)?”

Responses were given on a 7‐point scale with labeled endpoints (1 = not dangerous at all, 7 = very dangerous). An obvious disadvantage of the conditional measurement is that it is likely to be suggestive, overemphasizing the actual difference in risk perception between the two conditions. However, we expect this to happen equally in all experimental groups, thus not confounding the experimental design.

#### 2.4.2 Trust

Trust in state authorities of radiation protection was measured using five items adapted from the “trust in the scientific community” scale by Nisbet et al. (2015). Participants indicated agreement with statements such as: “Information from state authorities of radiation protection (e.g., the German/Greek radiation protection agency) is trustworthy.” Responses were captured on a 7‐point scale.

#### 2.4.3 Additional Variables

**Figure 2:**
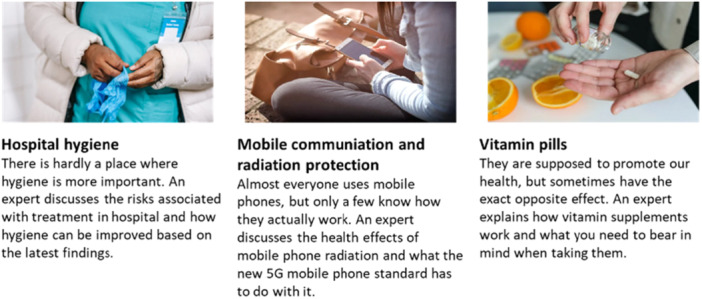
Articles for the question on practical relevance.

To allow exploratory analyses, the following constructs were also assessed:

- Practical relevance (how likely someone would show interest in a topic if coming across it in their daily life) of the topic “RF‐EMF and health,” measured via a click‐choice paradigm. Participants saw three article titles with brief descriptions, covering the topics “Mobile communication and radiation protection,” “Hospital hygiene,” and “Vitamin pills.” They indicated how likely they would be to click on each article during a waiting situation, 1 being “very unlikely” and 7 “very likely” (Figure 2).
- Thematic relevance (how often someone thinks about a topic) and discursive relevance (how often someone discusses a topic with other people) were assessed using the items: “How often in your daily life do you think about the topic ‘mobile phone radiation and health’?” and “How often in your daily life do you talk to others about the topic ‘mobile phone radiation and health’?” (1 = never, 7 = very often) (Wiedemann et al. 2017).
- “Self‐efficacy regarding precautionary behavior” was assessed with one item asking about confidence in one's ability to reduce personal RF‐EMF exposure.
- “Perceived text consistency,” measured by a single item.
- “Previous experience with precautionary behavior” and “knowledge about precautionary measures,” each measured by one item at the end of the survey.

### 2.5 Analysis Methods

To investigate the effects of information type (condition) on the dependent variables, one‐way analyses of variance (1×3 ANOVAs) were used. These analyses compared the three experimental conditions: (1) Basic information, (2) simple precautionary information, and (3) conceptual precautionary information. To first examine systematic differences in gender (female vs. male) and country (Germany vs. Greece), *t*‐tests were conducted. As systematic differences in gender and country emerged across several variables, both were also included as independent factors in the ANOVA models. To test Hypotheses *H*1 and *H*2 (comparing Group 1 and Group 2), and *H*3 and *H*4 (comparing Group 2 and Group 3), planned comparisons using Tukey's *t*‐tests for independent samples were performed. For variables where the assumption of homogeneity of variances was violated (in this case, affective risk perception), a Welch ANOVA was additionally conducted as a robustness check. The effect size (Cohen's *d*) was calculated to evaluate the size of differences between groups. The specific hypotheses, their corresponding dependent variables, comparison groups, and expected effects can be seen in Table 3.

**Table 3:** Summary of hypotheses and expected effects.

Hypothesis	Comparison groups	Dependent variable	Expected direction of effect
**H1a**	Basic information (Group 1) vs. simple precautionary information (Group 2)	Affective risk perception	Higher in Group 2
**H1b**	Group 1 vs. Group 2	CR1 (if no precautionary measures are taken)	Higher in Group 2
**H1c**	Group 1 vs. Group 2	CR2 (if precautionary measures are taken)	No difference expected
**H2**	Group 1 vs. Group 2	Trust in state authorities of radiation protection	Lower in Group 2
**H3a**	Simple precautionary information (Group 2) vs. conceptual precautionary information (Group 3)	Affective risk perception	Lower in Group 3
**H3b**	Group 2 vs. Group 3	CR1	Lower in Group 3
**H3c**	Group 2 vs. Group 3	CR2	No difference expected
**H4**	Group 2 vs. Group 3	Trust in state authorities of radiation protection	Higher in Group 3

## 3 Results

### 3.1 Data Preparation

Data analysis was conducted using JASP (version 0.19.3) and LibreOffice Calc. After downloading the raw dataset, participants not fulfilling the inclusion criteria were excluded (see Section 2). All items were re‐coded where necessary so that higher values always represented higher agreement or stronger perception. As affective risk perception was assessed with six items (three referring to mobile phones and three to base stations), internal consistency was tested and found to be very high (Cronbach's *α* = 0.97). The mean was calculated by averaging the six items to form one overall affective risk perception score. Conditional risk perception (CR1 and CR2) was assessed with one item each and therefore not aggregated. For the five items measuring trust in state authorities of radiation protection the internal consistency of the scale was good (Cronbach's *α* = 0.87), and a mean score was computed.

Before running inferential statistics, assumption checks were conducted. Normal distribution was tested using the Shapiro–Wilk test and was not confirmed for any of the dependent variables (all *p*‐values < 0.05). However, ANOVA is considered robust against violations of the normality assumption, especially in large samples such as the current one (*N* > 2000) (Eid et al. 2010; Knief and Forstmeier 2021). Homogeneity of variances was tested using Levene's test. It was fulfilled for all dependent variables except affective risk perception. Given the approximately equal group sizes and the robustness of ANOVA in large samples, the analysis was continued using one‐way ANOVAs. For affective risk perception, a Welch ANOVA was additionally computed as a robustness check.

### 3.2 Descriptive Results

#### 3.2.1 Country

Significant differences between participants from Germany and Greece were observed regarding risk perception, trust, and personal relevance of the topic (Tables 4 and 5). Risk perception and relevance were significantly higher, and trust was significantly lower in Greece compared to Germany.

**Table 4:** Country differences in practical, thematic, and discursive relevance.

Country	Practical relevance		Thematic relevance		Discursive relevance	
	Germany	Greece	Germany	Greece	Germany	Greece
Mean	*M* = 3.59	*M* = 4.93	*M* = 2.62	*M* = 3.97	*M* = 2.14	*M* = 3.4
SD	SD = 1.95	SD = 1.73	SD = 1.5	SD = 1.76	SD = 1.36	SD = 1.69
Difference	*t*(2167) = −17.04, *p* < 0.001, *d* = −0.73		*t*(2167) = −19.07, *p* < 0.001, *d* = −0.82		*t*(2167) = −18.99, *p* < 0.001, *d* = −0.82	

*Note: N* = 1040 in Germany, *N* = 1129 in Greece. Effect size measure: Cohen's *d*.

**Table 5:** Country differences in affective and conditional risk perception.

Country	Affective risk perception		CR1		CR2	
	Germany	Greece	Germany	Greece	Germany	Greece
Mean	*M* = 2.55	*M* = 4.3	*M* = 3.35	*M* = 4.92	*M* = 2.61	*M* = 3.66
SD	SD = 1.39	SD = 1.54	SD = 1.55	SD = 1.45	SD = 1.39	SD = 1.51
Difference	*t*(2167) = −27.69, *p* < 0.001, *d* = −1.19		*t*(2167) = −24.28, *p* < 0.001, *d* = −1.04		*t*(2167) = −16.94, *p* < 0.001, *d* = −0.73	

*Note: N* = 1040 in Germany, *N* = 1129 in Greece. Scales on affective RP ranged from 1 (not at all) to 7 (very much), on conditional RP from 1 (not dangerous at all) to 7 (very dangerous), on trust from 1 (do not agree at all) to 7 (totally agree). Effect size measure: Cohen's *d*.

Furthermore, in Greece, significantly more participants than in Germany indicated that they would read the articles on the other two health‐related topics hospital hygiene (Greece: *M* = 4.41, SD = 1.93; Germany: *M* = 3.87, SD = 2.02; *t*(2167) = −6.36, *d* = 0.27) and vitamin pills (Greece: *M* = 4.67, SD = 1.91; Germany: *M* = 4.04, SD = 2.06; *t*(2167) = −7.43, *d* = 0.32). However, differences were not as big as for the article on RF‐EMF and health.

The proportion of participants who answered “yes” or “partly” to the questions if they had already implemented measures to reduce their personal exposure to RF‐EMF when using mobile devices in their daily lives and if they had already been aware that the precautionary measures described could reduce personal exposure to RF‐EMF (only Groups 2 and 3) was higher in Greece than in Germany (Table 6).

**Table 6:** Responses to the questions on prior experiences with precautionary measures and information.

	Already implemented measures		Were aware of measures	
	Germany	Greece	Germany	Greece
Yes	124 (11.92%)	219 (19.4%)	197 (18.94%)	327 (32.06%)
Partly	431 (41.44%)	675 (59.79%)	318 (30.58%)	363 (32.15%)
No	484 (46.54%)	234 (20.73%)	176 (16.92%)	77 (6.83%)
Missing	1 (0.1%)	1 (0.1%)		
Basic text group			349 (33.56%)	362 (32.06%)

#### 3.2.2 Gender

In Germany, gender differences were found in affective risk perception, general conditional risk perception assuming that no precautions are taken (CR1), and thematic relevance, with females reporting higher risk perception and relevance than males. In Greece, gender differences were found in all risk perception measures and thematic relevance, with females reporting higher risk perception and higher relevance than males. Additionally, there was a significant difference in trust, with males reporting higher trust than females (Table 7).

**Table 7:** Gender differences.

	Total female	Total male	Germany	Germany	Greece	Greece
	(*n* = 1107)	(*n* = 1062)	female	male	female	male
			(*n* = 532)	(*n* = 508)	(*n* = 575)	(*n* = 554)
Affective RP	*M* = 3.66	*M* = 3.26	*M* = 2.68	*M* = 2.42	*M* = 4.56	*M* = 4.03
	SD = 1.71	SD = 1.69	SD = 1.4	SD = 1.36	SD = 1.46	SD = 1.59
	*t*(2167) = 5.47, *p* < 0.001, *d* = 0.24		*t*(1038) = 3.12, *p* = 0.002, *d* = 0.19		*t*(1127) = 5.83, *p* < 0.001, *d* = 0.35	
CR1	*M* = 4.42	*M* = 3.9	*M* = 3.57	*M* = 3.13	*M* = 5.21	*M* = 4.61
	SD = 1.67	SD = 1.67	SD = 1.56	SD = 1.5	SD = 1.36	SD = 1.49
	*t*(2167) = 7.29, *p* < 0.001, *d* = 0.31		*t*(1038) = 4.65, *p* < 0.001, *d* = 0.29		*t*(1127) = 7.13, *p* < 0.001, *d* = 0.42	
CR2	*M* = 3.33	*M* = 2.98	*M* = 2.71	*M* = 2.5	*M* = 3.89	*M* = 3.43
	SD = 1.56	SD = 1.51	SD = 1.38	SD = 1.4	SD = 1.5	SD = 1.48
	*t*(2167) = 5.2, *p* < 0.001, *d* = 0.22		*t*(1038) = 2.51, *p* = 0.01, *d* = 0.16		*t*(1127) = 5.24, *p* < 0.001, *d* = 0.32	
Trust	*M* = 4.32	*M* = 4.5	*M* = 4.62	*M* = 4.78	*M* = 4.04	*M* = 4.25
	SD = 1.33	SD = 1.43	SD = 1.37	SD = 1.42	SD = 1.22	SD = 1.39
	*t*(2167) = −3.12, *p* = 0.002, *d* = −0.13		*t*(1038) = ‐1.83, *p* = 0.07, *d* = −0.11		*t*(1127) = ‐2.71, *p* = 0.01, *d* = −0.16	
Practical relevance	*M* = 4.22	*M* = 4.36	*M* = 3.47	*M* = 3.72	*M* = 4.91	*M* = 4.94
	SD = 1.98	SD = 1.9	SD = 1.95	SD = 1.87	SD = 1.73	SD = 1.72
	*t*(2167) = −1.7, *p* = 0.09, *d* =−0.07		*t*(1038) = −2.13, *p* = 0.03, *d* = 0.13		*t*(1127) = −0.32, *p* = 0.75, *d* = −0.02	
Thematic relevance	*M* = 3.4	*M* = 3.26	*M* = 2.65	*M* = 2.59	*M* = 4.01	*M* = 3.83
	SD = 1.79	SD = 1.76	SD = 1.5	SD = 1.49	SD = 1.75	SD = 1.77
	*t*(2167) = 2.21, *p* = 0.03, *d* = 0.01		*t*(1038) = 0.69, *p* = 0.49, *d* = 0.04		*t*(1127) = 2.58, *p* = 0.01, *d* = 0.15	
Discursive relevance	*M* = 2.77	*M* = 2.83	*M* = 2.09	*M* = 2.2	*M* = 3.4	*M* = 3.4
	SD = 1.66	SD = 1.67	SD = 1.34	SD = 1.38	SD = 1.68	SD = 1.71
	*t*(2167) = 0.75, *p* = 0.46, *d* = 0.04		*t*(1038) = −1.22, *p* = 0.22, *d* = −0.08		*t*(1127) = −0.26, *p* = 0.98, *d* = −0.002	

#### 3.2.3 Age

Regarding age, we differentiated between five age groups: 18–29, 30–39, 40–49, 50–59, and 60+. For the sake of readability, detailed comparisons between these age groups are not shown here but can be found online under https://osf.io/p9whn/. Summarized, in Germany, there were significant age differences for affective risk perception and general conditional risk perception assuming that precautions are taken (CR2). Post‐hoc Tukey tests revealed that these differences were mostly due to the oldest age group (60+) indicating lower risk perception than some of the younger age groups. Also, thematic and discursive relevance were lower in the 60+ group compared to the 18–29 and 40–49 groups. The age differences regarding trust can be attributed to higher trust in the youngest age group (18–29) compared to some of the older ones. In Greece, there were significant age differences for affective risk perception and both conditional risk perception measures, with post‐hoc tests showing that the youngest age group indicated lower risk perception than all the older ones. Furthermore, practical, thematic and discursive relevance were lower in the 18–29 group compared to almost all others. While in Germany, the oldest age group indicated lower risk perception and relevance of the topic compared to a part of the younger ones, in Greece it was the youngest age group that generally reported lower risk perception and relevance.

### 3.3 Relations Between Variables

To examine relationships between the central variables, Pearson correlation coefficients were calculated (Table 8). Due to the large sample sizes, most correlations are statistically significant even when small. Affective and conditional risk perception measures were strongly correlated and positively associated with all three relevance measures. This suggests that higher risk perception tends to go along with perceiving the topic as more relevant. Trust was negatively correlated with all risk perception measures as well as thematic and discursive relevance, indicating that lower trust is associated with higher perceived risk and relevance of the topic. Generally, the correlations were similar in Germany and Greece, except for the association between the relevance variables and trust. Practical relevance and trust did not correlate significantly in Germany (*r* = ‐0.01, *p* = 0.7), but in Greece (*r* = 0.09, *p* = 0.002). Thematic and discursive relevance correlated significantly with trust in Germany (thematic: *r* = 0.09, *p* = 0.002; discursive: *r* = −0.15, *p* < 0.001), but not in Greece (thematic: *r* = −0.02, *p* = 0.45; discursive: *r* = 0.01, *p* = 0.73).

**Table 8:** Correlations among risk perception, trust, and perceived relevance (total sample).

	CR1	CR2	Trust	Practical relevance	Thematic relevance	Discursive relevance
Affective RP	*r* = 0.79	*r* = 0.69	*t* = −0.29	*r* = 0.51	*r* = 0.69	*r* = 0.64
	*p* < 0.001	*p* < 0.001	*p* < 0.001	*p* < 0.001	*p* < 0.001	*p* < 0.001
CR1		*r* = 0.68	*r* = −0.22	*r* = 0.46	*r* = 0.58	*r* = 0.51
		*p* < 0.001	*p* < 0.001	*p* < 0.001	*p* < 0.001	*p* < 0.001
CR2			*r* = −0.3	*r* = 0.36	*r* = 0.51	*r* = 0.49
			*p* < 0.001	*p* < 0.001	*p* < 0.001	*p* < 0.001
Trust				*r* = −0.03	*r* = −0.15	*r* = −0.13
				*p* = 0.11	*p* < 0.001	*p* < 0.001
Practical relevance					*r* = 0.62	*r* = 0.56
					*p* < 0.001	*p* < 0.001
Thematic relevance						*r* = 0.8
						*p* < 0.001

To further analyze the high relation between thematic relevance and affective risk perception, participants were divided into three groups each (low, medium, and high thematic relevance/affective risk perception) (Table 9), following the analysis of previous studies (Wiedemann et al. 2017). Most participants who indicated low thematic relevance also had lower affective risk perception; however, this was more pronounced in Germany. In Greece, almost half of the participants who indicated a low thematic relevance, still perceived the risk to be medium or high. The same tendency can be seen for medium and high thematic relevance: 51% of the Greek participants reporting medium thematic relevance (compared to only 14% of the Germans) showed high affective risk perception. Almost 80% of the Greek participants who reported high thematic relevance also perceived the risk to be high, while this was the case for just under 50% of the Germany.

**Table 9:** Thematic relevance and affective risk perception.

	Low affective RP(*M* = 1.0–3.4)	Medium affective RP(*M* = 3.5–4.4)	High affective RP(*M* = 4.5–7.0)
Low thematic relevance (1, 2, 3)	Germany: 656 (85.4%)	Germany: 78 (10.2%)	Germany: 34 (4.4%)
	Greece: 241 (53.0%)	Greece: 111 (24.4%)	Greece: 103 (22.6%)
Medium thematic relevance (4)	Germany: 75 (51.7%)	Germany: 50 (34.5%)	Germany: 20 (13.8%)
	Greece: 41 (19.1%)	Greece: 64 (29.8%)	Greece: 110 (51.2%)
High thematic relevance (5, 6, 7)	Germany: 38 (29.5%)	Germany: 27 (20.9%)	Germany: 64 (49.6%)
	Greece: 31 (6.7%)	Greece: 64 (13.9%)	Greece: 365 (79.3%)

### 3.4 Hypothesis Tests

To examine the effects of the different types of precautionary information, ANOVAs and post‐hoc tests were conducted. First, Groups 1 (basic text) and 2 (simple precautionary information) were compared regarding their risk perception (*H*1) and trust (*H*2). Then, Groups 2 (simple precautionary information) and 3 (conceptual precautionary information) were compared, also regarding their risk perception (*H*3) and trust (*H*4). Country and gender were included in the analyses as additional factors due to the previously identified group differences. Table 10 shows the means and standard deviations for the dependent variables that are relevant for the following analyses.

**Table 10:** Means and standard deviations for dependent variables.

Group	1	2	3	1	2	3
	**Affective risk perception**			**CR1**		
Mean	*M* = 3.31	*M* = 3.44	*M* = 3.61	*M* = 3.9	*M* = 4.26	*M* = 4.34
SD	SD = 1.72	SD = 1.68	SD = 1.72	SD = 1.72	SD = 1.66	SD = 1.66
	**CR2**			**Trust**		
Mean	*M* = 3.05	*M* = 3.14	*M* = 3.27	*M* = 4.44	*M* = 4.42	*M* = 4.37
SD	SD = 1.54	SD = 1.52	SD = 1.58	SD = 1.42	SD = 1.36	SD = 1.37
	**Self‐efficacy**			**Consistency**		
	*M* = 4.54	*M* = 5.08	*M* = 5.09	*M* = 5.05	*M* = 5.34	*M* = 5.36
	SD = 1.56	SD = 1.42	SD = 1.44	SD = 1.31	SD = 1.26	SD = 1.26

*Note:* Group 1 = Basic text (*N* = 711), Group 2 = simple precautionary information (*N* = 727), Group 3 = conceptual precautionary information (*N* = 731). Scales on affective RP ranged from 1 (not at all) to 7 (very much), on conditional RP from 1 (not dangerous at all) to 7 (very dangerous), on trust, self‐efficacy, and consistency from 1 (do not agree at all) to 7 (totally agree).

#### 3.4.1 Analyses on Affective Risk Perception

The analysis on affective risk perception revealed significant main effects for condition (*F* (2, 2157) = 6.19, *p* = 0.002, *η*_p_^2^ = 0.006), gender (higher in females, *F* (1, 2157) = 41.67, *p* < 0.001, *η*_p_^2^ = 0.019), and country (higher in Greece, *F* (1, 2157) = 770.49, *p* < 0.001, *η*_p_^2^ = 0.263). Post‐hoc tests showed that affective risk perception was significantly lower in Group 1 (basic text) than in Group 3 (conceptual precautionary information) (*t* (2157) = −3.51, *p* = 0.001, *d* = −0.19). These effects remained significant when analyses were performed separately for each country. However, there were no significant differences between Group 1 and Group 2 or Group 2 and Group 3. Thus, H1a and H3a were not supported.

**Figure 3:**
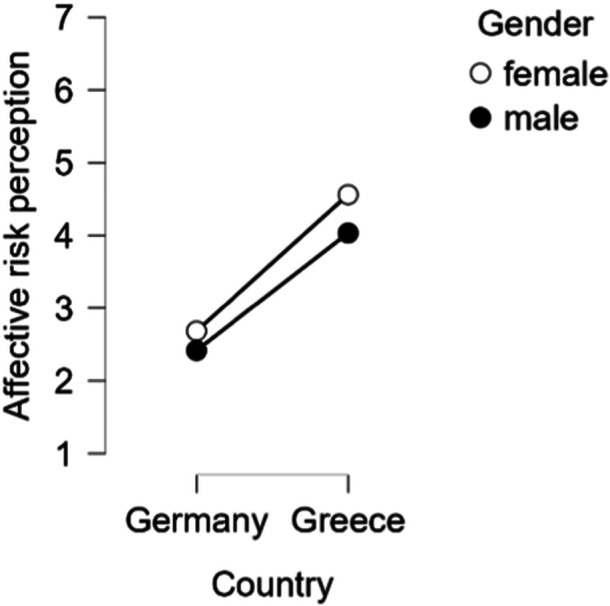
Interaction between gender and country on affective risk perception.

**Figure 4:**
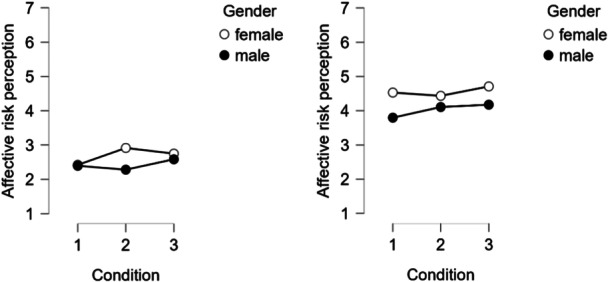
Left: Germany. Right: Greece. Three‐way interaction between condition, gender, and country on affective risk perception.

A significant interaction between gender and country was observed (Figure 3), indicating that gender differences were more pronounced in Greece than in Germany, *F* (1, 2157) = 4.26, *p* = 0.039, *η*_p_^2^ = 0.002). Additionally, a significant three‐way interaction (Figure 4) between condition, gender, and country emerged (*F* (2, 2157) = 5.69, *p* = 0.003, *η*_p_^2^ = 0.005). Follow‐up analyses revealed that in Germany, a two‐way interaction between condition and gender was significant, with the largest gender difference found in Group 2. In contrast, this interaction was not significant in Greece, where gender differences were more stable across conditions.

To check the robustness of the main effect despite the violation of normality and homogeneity assumptions, a Welch‐ANOVA was additionally performed for the factor “text condition.” The results were consistent with those from the standard ANOVA.

#### 3.4.2 Analyses on CR1

A factorial ANOVA was conducted to examine the effects of text condition, gender, and country on conditional risk perception assuming that no precautions are taken (CR1). The analysis revealed significant main effects for condition (*F* (2, 2157) = 17.56, *p* < 0.001, *η*_p_^2^ = 0.016), gender (higher in females, *F* (1, 2157) = 72.9, *p* < 0.001, *η*_p_^2^ = 0.033), and country (higher in Greece, *F* (1, 2157) = 605.33, *p* < 0.001, *η*_p_^2^ = 0.219). Post‐hoc comparisons showed that participants in Group 1 (basic text) reported significantly lower conditional risk perception (assuming that no precautions are taken) than those in Group 2 (simple precautionary information, *t* (2157) = −5.08, *p* < 0.001, *d* = −0.27) and Group 3 (conceptual precautionary information *t* (2157) = −5.22, *p* < 0.001, *d* = −0.28). These effects remained significant when analyses were performed separately for each country.

**Figure 5:**
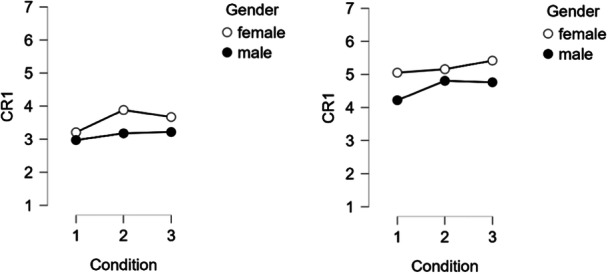
Left: Germany. Right: Greece. Three‐way interaction between condition, gender, and country on CR1.

A significant three‐way interaction (Figure 5) between condition, gender, and country was found (*F* (2, 2157) = 10.28, *p* = 0.008, *η*_p_^2^ = 0.004). Follow‐up analyses indicated that the gender difference was largest in Group 2 in Germany, while in Greece, it was more pronounced in Groups 1 and 3. This pattern is consistent with that of the three‐way interaction effect for affective risk perception.

#### 3.4.3 Analyses on CR2

**Figure 6:**
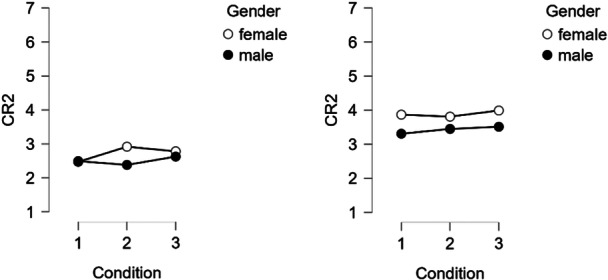
Left: Germany. Right: Greece. Three‐way interaction between condition, gender, and country on CR2.

For conditional risk perception assuming that precautions are taken (CR2), the hypotheses had been that there would be no significant group differences. In the ANOVA, significant main effects were found for condition (*F* (2, 2157) = 3.2, *p* = 0.04, *η*_p_^2^ = 0.003), gender (higher in females, *F* (1, 2157) = 30.97, *p* < 0.001, *η*_p_^2^ = 0.014), and country (higher in Greece, *F* (1, 2157) = 284.03, *p* < 0.001, *η*_p_^2^ = 0.116). Post‐hoc tests showed that conditional risk perception (assuming that precautions are taken) was significantly lower in Group 1 (basic text) than in Group 3 (conceptual precautionary information), *t* (2157) = −2.53, *p* = 0.03, *d* = −0.13). No significant differences were found between Group 2 and the other groups. While the gender effect was consistent across both countries, the effect of condition was not: It was only marginally significant when countries were analyzed separately. A significant three‐way interaction (Figure 6) between condition, gender, and country was found (*F* (2, 2157) = 3.21, *p* = 0.04, *η*_p_^2^ = 0.003). Further analyses showed that this interaction was primarily driven by a significant gender × condition interaction in Germany, with the most pronounced gender difference found in Group 2. In Greece, this interaction was not significant. This mirrors the pattern observed for affective risk perception and conditional risk perception assuming that no precautions are taken (CR1).

#### 3.4.4 Analyses on Trust

**Figure 7:**
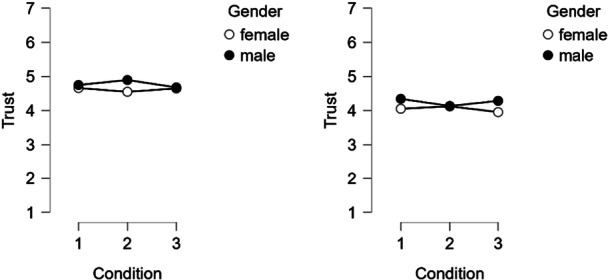
Left: Germany. Right: Greece. Three‐way interaction between condition, gender, and country on trust.

In the ANOVA for trust, no significant main effect was found for condition (Figure 7). Significant main effects were found for gender (lower in females, *F* [1, 2157] = 9.87, *p* = 0.002, *η*_p_^2^ = 0.005), and country (lower in Greece, *F* (1, 2157) = 89.01, *p* < 0.001, *η*_p_^2^ = 0.040). None of the interaction effects were significant.

### 3.5 Further Exploratory Analyses

#### 3.5.1 Self‐Efficacy and Perceived Consistency

**Figure 8:**
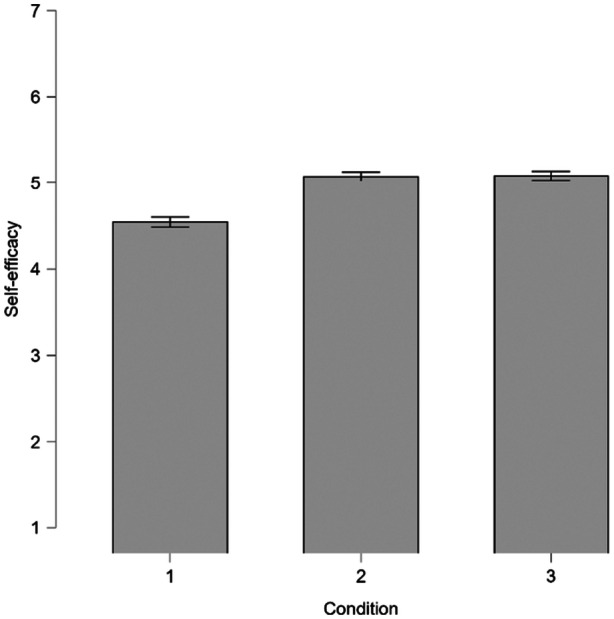
Main effect of condition for self‐efficacy (standard errors).

To explore possible mechanisms behind the effects of the texts, participants were asked about their feeling of self‐efficacy after reading the information and how consistent they perceived the information to be. In the ANOVA for self‐efficacy, there was a significant main effect of condition (*F* (2, 2157) = 32.13, *p* < 0.001, *η*_p_^2^ = 0.029). Post‐hoc comparisons showed that participants in Group 1 (basic text) reported lower self‐efficacy than those in Group 2 (simple precautionary information), (*t* (2157) = −6.95, *p* < 0.001, *d* = −0.37) and Group 3 (conceptual precautionary information) (*t* (2157) = −6.97, *p* < 0.001, *d* = 0.37) (Figure 8).

**Figure 9:**
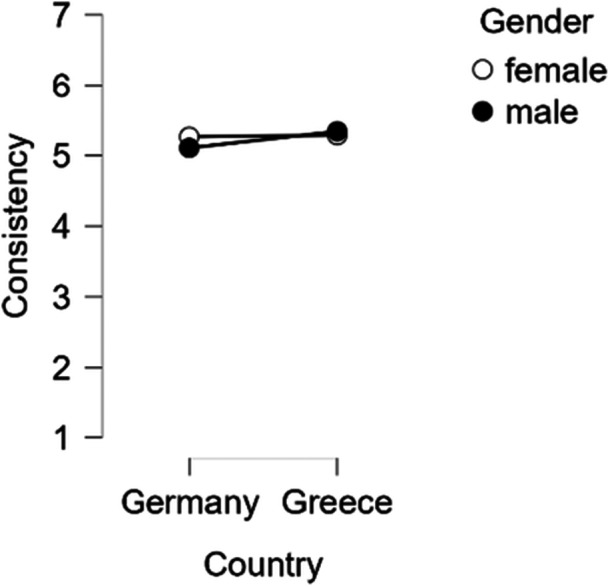
Interaction between gender and country on perceived consistency.

In the ANOVA for perceived consistency, there were significant main effects of condition (*F* (2, 2157) = 13.51, *p* < 0.001, *η*_p_^2^ = 0.012) and country (higher in Greece, *F* (1, 2157) = 5.06, *p* = 0.03, *η*_p_^2^ = 0.002). Post‐hoc tests showed that participants in Group 1 perceived the information as less consistent than those in Group 2 (*t* (2157) = −4.36, *p* < 0.001, *d* = −0.23) and Group 3 (*t* (2157) = −4.65, *p* < 0.001, *d* = −0.25). Furthermore, a significant interaction between gender and country was found (*F* (2, 2157) = 4.22, *p* = 0.04, *η*_p_^2^ = 0.002), which was driven by a difference between German and Greek males: German males rated consistency lower than Greek males (*t* (2157) = −3.01, *p* = 0.01, *d* = −0.19) (Figure 9).

## 4 Discussion

### 4.1 Summary and Discussion of the Main Findings

The present study investigated effects of different kinds of precautionary information on risk perception and trust, including gender and country as other potential influencing factors. The main results are summarized in Table 11.

**Table 11:** Summary of the analysis.

Variable	Country	Gender	Condition	2‐way interactions	3‐way interactions
Affective risk perception	Greece > Germany	Females > males	Group 3 > Group 1	Gender × Country, (stronger difference in Greece)	3‐way interaction (only Germany: Significant gender difference in condition 2)
CR1	Greece > Germany	Females > males	Groups 2 and 3 > Group 1	—	3‐way interaction (only Germany: Significant gender difference in condition 2)
CR2	Greece > Germany	Females > males	Group 3 > Group 1	—	3‐way interaction (only Germany: Significant gender difference in condition 2)
Trust	Germany > Greece	Males > Females	—	—	—
Self‐Efficacy	—	—	Groups 2 and 3 > Group 1		—
Consistency	Greece > Germany	—	Groups 2 and 3 > Group 1	Gender × Country (stronger differences between males)	

#### 4.1.1 Effects of Different Precautionary Information

One objective of this study was to investigate if simple precautionary information referring to personal mobile phone use leads to an increase in perceived risk and to lower trust compared to basic information without any precautionary content. This question was of interest because while a number of previous studies had found these effects (e.g., Boehmert et al. 2020), others had not (Boehmert et al. 2016; Eggeling‐Böcker et al. 2025). In the present study, simple precautionary information did neither lead to significantly higher affective risk perceptions nor to lower trust compared to the basic text. Only when participants judged the risk under the condition that no precautions are taken, those in the simple precautionary information group indicated higher values than those in the basic information group. Moreover, participants who received precautionary information (simple or conceptual) perceived the information as more consistent and reported higher self‐efficacy compared to those who received only the basic text. This is noteworthy because prior research has discussed (Boehmert et al. 2016) and reported qualitative evidence (Timotijevic and Barnett 2006) that perceived inconsistency of messages including precautionary information could be a mediator variable in the increase of risk perception due to precautionary information. The current study was the first to measure perceived consistency quantitatively. Apparently, in this study, precautionary information did not generally trigger worries or doubts and did also have no negative effects on trust.

As mentioned before, this is not the first study finding no or few general effects of precautionary information: Recent studies (Boehmert et al. 2016; Eggeling‐Böcker et al. 2025) have found few general differences between precautionary and basic information only for part of the variables or only if considering individual differences like trait anxiety and gender. This may be explained by the fact that researchers used different kinds of precautionary information: Earlier studies often presented institutional precautionary information (e.g., precautionary limit values), while more recent studies mostly used personal precautionary information (referring to measures one can take individually to reduce RF‐EMF exposure when using mobile devices). It is well possible that personal precautionary information is perceived as more helpful and is more likely to strengthen self‐efficacy. Institutional information on the other hand may leave individuals feeling at the mercy of other people's decisions, which could go along with a higher risk perception.

The other objective of this study was to assess whether conceptual precautionary information would lead to lower risk perception and higher trust compared to simple precautionary information. While there would be many ways to make precautionary information more detailed or explain the concept further, we decided to investigate whether an explanation of the difference between the concepts “precaution” and “prevention” would make a difference. The idea behind that was that recipients may perceive information about ways to lower their exposure to RF‐EMF when using a mobile phone as inconsistent when they are also told that there is no proven harm of RF‐EMF to human health under the limit values in place. This can result in higher risk perception and lower trust in authorities communicating this information, which has been the case in previous studies (Boehmert et al. 2020). The general idea behind adding more information on the concept of precaution was that this could lead to a better understanding of communication intent and consequently weaken the expected negative effect of precautionary information on risk perception and trust compared to basic information. The additional information in the group receiving conceptual precautionary information, however, did not have the intended effect: It didn't lead to lower risk perception or higher trust. All comparisons between simple and conceptual precautionary information yielded insignificant results, hence not supporting our hypotheses. However, exploratory comparisons between conceptual precautionary information and basic information yielded significant results. Affective risk perception and conditional risk perception were all significantly higher after the conceptual precautionary information compared to the basic information.

In the following, we draw practical conclusions from the perspective of a risk communicator that has the goal to communicate precautionary information but at the same time does not want to increase public risk perception about RF‐EMF from mobile communications. Taking the results of the hypothesis tests and the exploratory analysis into account, providing the conceptual explanation of the difference between precaution and prevention is very likely an inadequate way of achieving these goals and in fact might even backfire. In this study, all three risk perception measures were highest in the group receiving the conceptual information, with the differences to the group that received no precautionary information at all being significant. Reasons for this finding are not obvious. It is possible that the additional information led to a more in‐depth examination of the topic by the participants, which in turn led to an increase in perceived risk. This interpretation fits in with the survey processing times. As would be expected, the time spent on the survey was higher in the conceptual precautionary information group (approximately 9 min) than in the simple precautionary information group (approximately 8 min) and in the basic information group (approximately 6.5 min). It thus might be that not the precautionary information as the content, but rather the amount of information given led to more thorough information processing, thereby elevating risk perceptions. It also could be that (some) participants applied a heuristic, taking the amount of information given as an indicator of the seriousness of the issue at hand. Additional explanations around precautionary information have been found to increase risk perception before (Boehmert et al. 2016). In that study, information explaining the effectiveness of precautionary measures led to higher risk perception under the condition that no precautions are taken (CR1). Based on that study and the current results, it seems better to keep precautionary information simple instead of adding a lot of explanations.

In some cases, interaction effects showed that reactions to different information varied depending on the interplay of country, gender, and information type. Explanations for these findings are not obvious. Possibly, there are elements in the information texts that affect males and females in specific ways. Future studies could usefully investigate this issue further.

#### 4.1.2 Effects of Country and Gender

There were sociodemographic differences for risk perception and trust as well as for personal relevance of the topic. Females reported higher risk perception and relevance of the topic, and lower trust than males in both countries. The findings also revealed that the gender differences were in some cases more pronounced in Greece. However, it needs to be considered that mean differences were quite small. Generally, it is in line with previous research that females show higher levels of RF‐EMF risk perception and relevance and lower trust than males (Davidson and Freudenburg 1996).

Regarding country, significant differences between Germany and Greece were found, with higher risk perception and lower trust observed in Greece. Practical, thematic, and discursive relevance of the topic were also significantly higher in Greece compared to Germany, indicating that Greek citizens think and talk more about it in their daily lives compared to Germans. Thinking about the topic regularly and perceiving it as risky was highly correlated. Particularly in Greece, those who perceived the thematic relevance of the topic as medium or high did also report medium or high affective risk perception. This is in line with previous findings, as RF‐EMF from mobile communications and institutions that communicate about it have already been found to be viewed more critically in Greece compared to Germany (TNS Opinion & Social 2010). Interestingly, in our study, interest in the two other health‐related topics in the question on practical relevance was also higher in Greece, indicating more interest in health‐related issues in general. However, as a Likert‐scaled survey was used to capture responses, it can't be ruled out that the Greek participants just generally tended to give more extreme answers in our questionnaire.

#### 4.1.3 Implications for Research and Risk Communication

Generally, the findings from this study may be relevant for authorities communicating precautionary information or considering doing so. The results, unlike some earlier studies, suggest that simple precautionary information does not necessarily lead to higher risk perception and lower trust, and could even increase the feeling of self‐efficacy. Even though it hasn't been experimentally compared, it may be important which kind of precautionary information (personal or not?) is used and how it is explained. If recipients feel that the information is constructive and gives them (some) control over their personal exposure by RF‐EMF from mobile communications, they may feel more self‐ efficacious and perceive the information as more consistent and possibly trustworthy. However, results from this study also suggest that adding information on the concept of precaution can lead to higher risk perception and should be considered with caution.

Regarding trust, it needs to be considered that in this study a fictional institution was the source of information; in real life, existing attitudes about communicating institutions may influence the perception of information by this source. Trust in existing institutions is unlikely to be based only on one piece of information, but also on previous experiences. Regarding sociodemographic differences, the findings from this study highlight that a nuanced approach to risk communication may be sometimes useful, particularly when addressing diverse audiences, for example from different countries. Targeting information to specific demographics, such as gender, cultural background, and prior risk perceptions, could be helpful. Especially, in countries with higher general risk perception regarding the topic, such as Greece, public health messaging may benefit from explaining precautionary measures with clear, concise, and easily digestible information that empowers individuals without overwhelming them.

### 4.2 Limitations

The present study has several limitations that must be considered when interpreting the results and drawing conclusions, some of which have already been mentioned. Regarding the data collection methods, the study relied on self‐report‐measures, which may be subject to biases, for example social desirability, and used Likert‐response scales, which can be subject to response tendencies. Also, due to the quantitative and cross‐sectional design, individual changes were not measured: The observed impacts may consequently be only short‐term, and there is no way of knowing why participants reacted in certain ways. Long‐term studies or qualitative research would be necessary to gain a deeper understanding. Regarding the sample, although we were able to recruit a heterogenous sample with interlocking age and gender quotas, it was of course not a random sample drawn from the general population. Also, during data collection, not enough participants from the older age groups in Greece (particularly females) were recruited, so these groups are underrepresented in the sample. Generally, the study focused on only two countries, and on RF‐EMF from mobile communications (particularly mobile phones), limiting the findings to Germany and Greece and to this particular source of RF‐EMF. Future research should aim to address these limitations and further explore the factors influencing risk perception and trust in different sociocultural contexts.

One needs to keep in mind though that most of the effects found in this study were small and the explained variance low, indicating that they should not be over‐interpreted and that other aspects are more important for differences in risk perception and trust.

## 5 Conclusions

While risk communicators need to weigh the arguments for or against the communication of precautionary information regarding RF‐EMF from mobile communications, it was the aim of the current study to shed more light on the effects that communicating precaution has on message recipients.

In that sense, this study provides insights into the influence of different types of precautionary information on risk perception and trust in radiation protection authorities regarding RF‐EMF from mobile communications, especially 5G NR. This study specifically compared responses from participants in Germany and Greece. One important finding of this study is that simple precautionary information did not lead to higher affective risk perception and lower trust. In the case of risk perception, this finding contrasts with the results of a meta‐analysis (Boehmert et al. 2020). Only risk perception under the explicit condition that no precautionary measures are taken was higher. Furthermore, simple precautionary information resulted in higher self‐efficacy and perceived consistency of the information. This may be due to different kinds of precautionary information used (institutional information in older studies, recommendation referring to personal mobile phone use in this and more recent studies).

The additional conceptual explanation of precaution compared to prevention did not have the intended effect of lowering risk perception and increasing trust. Compared to the basic text group, it even led to higher affective risk perception and conditional risk perceptions. This suggests that there is no advantage in adding longer, more theoretical explanations of the concept and that simple, practical recommendations may be more helpful.

There were some unexpected interaction effects on the risk perception variables which indicate that reactions to different types of information may vary depending on the interplay of country, gender, and information type. However, effect sizes were generally small and such findings should not be overinterpreted.

In line with previous research, sociodemographic differences were found for risk perception and trust as well as for personal relevance of the topic. RF‐EMF from mobile communications and authorities communicating about it are viewed more critically in Greece compared to Germany. In line with past studies, risk perception and relevance were higher, and trust was lower in this study as well. In both countries, females showed higher risk perception and relevance and lower trust than males.

This study suggests that precautionary information may be communicated effectively without increasing public concern, if given in form of practical, feasible recommendations. If this can be confirmed by other studies and in practice, precautionary information can be given by risk communicators without causing unnecessary alarm. Even if not completely transferrable, the findings may still be helpful to consider in other situations where new technologies (or developments of technologies) are introduced, for example new generations of mobile technology.

Summarized, our results suggest that precautionary information about RF‐EMF in mobile communications can be communicated without increasing public concerns or undermining trust in authorities, that clear and concise messages may be more helpful than detailed explanations, and that there are sociodemographic and individual differences in the perception of risk‐related information.

## Nomenclature

**Glossary**
5G NR: 5G New Radio
RF‐EMF: Radio‐frequency Electromagnetic Fields
GHz: Gigahertz
kHz: Kilohertz
WHO: World Health Organization
IARC: International Agency for Research on Cancer
ICNIRP: International Commission for Non‐Ionizing Radiation Protection
IEEE: Institute of Electrical and Electronic Engineers
BfS: Bundesamt fuer Strahlenschutz (German Federal Office for Radiation Protection)
EEAE: Greek Atomic Energy Commission (Greek national radiation protection authority)
CR1: (General) Conditional risk perception assuming that no precautions are taken
CR2: (General) Conditional risk perception assuming that precautions are taken

## Ethics Statement

All procedures were performed in compliance with relevant laws and institutional guidelines and have been approved in form of an ethics application (01/12/2023) by the committee of the IU International University of Applied Sciences before data collection started.

## Consent

The privacy of human subjects was observed and informed consent was obtained before participants began the experiment.

## Conflicts of Interest

The authors declare no conflicts of interest.

## Data Availability

Data and supplementary material are available on the Open Science Framework (OSF, https://osf.io/p9whn/. The project contains the following supplementary files:
− Data: Codebook for the study, data files (data in standard tabular format and statistical analyses in JASP‐format), age tables (detailed comparisons for both countries). − Experimental material: Information texts, complete survey, comparison of two studies (Eggeling‐Böcker et al. 2025 and this one). The study was pre‐registered prior to data collection under https://osf.io/b4cms.
